# Psychological Distress, Somatic Symptom Burden, and Quality of Life in Rheumatoid Arthritis: A Cross-Sectional Study in Bosnia and Herzegovina

**DOI:** 10.3390/healthcare14193179

**Published:** 2026-09-25

**Authors:** Asmir Konjic, Rusmir Softic, Renata Hodzic, Srebrenko Prcic, Mirela Hudic Begic

**Affiliations:** 1 Department of Physical Medicine and Rehabilitation, University Clinical Center Tuzla, 75000 Tuzla, Bosnia and Herzegovina; asmir.konjic@gmail.com (A.K.); srebro79@gmail.com (S.P.); begichudic@gmail.com (M.H.B.); 2 Department of Psychiatry, University Clinical Center Tuzla, 75000 Tuzla, Bosnia and Herzegovina; 3 Department of Neurology, University Clinical Center Tuzla, 75000 Tuzla, Bosnia and Herzegovina; renatahodzic1973@gmail.com

**Keywords:** rheumatoid arthritis, psychological distress, depression, anxiety, somatic symptoms, quality of life, rehabilitation

## Abstract

**Highlights:**

**What are the main findings?**

- Continuous psychological symptom scores were associated with poorer MANSA quality of life in exploratory analyses. Causal and diagnostic conclusions cannot be drawn.
- Somatic symptom scores were associated with pain and disability, but PHQ-15 content overlaps with manifestations of RA and chronic illness.

**What are the implications of the main findings?**

- Use of locally validated psychological and somatic assessment tools may complement rheumatologic care when linked to appropriate clinical evaluation and referral pathways.
- Larger longitudinal multicenter studies should test temporal relationships and generalizability beyond clinically burdened tertiary care populations.

**Abstract:**

Background: Rheumatoid arthritis (RA) affects physical and psychological well-being, but Southeast European evidence is limited. Methods: This cross-sectional study included 85 consecutive adults with RA at a tertiary rehabilitation center. Participants completed Bosnian translations of the PHQ-9, GAD-7, PHQ-15, Brief Symptom Inventory, and MANSA. Same-day assessment included pain VAS, DAS28-CRP(3), and HAQ-DI. Continuous scores were analyzed using 20 Spearman correlations with FDR adjustment and four exploratory MANSA models with HC3-robust standard errors. Four internally discordant GSI records were excluded from primary GSI analyses (*n* = 81). Post hoc sensitivity analyses included all 85 records. Results: Mean DAS28-CRP(3), HAQ-DI, and MANSA scores were 5.31, 1.69, and 4.37. MANSA correlated with PHQ-9 (rho = −0.49), GAD-7 (−0.30), PHQ-15 (−0.37), and GSI (−0.53; all q ≤ 0.016). PHQ-15 correlated with pain (rho = 0.36) and HAQ-DI (0.31), PHQ-9 correlated with HAQ-DI (0.25), and GSI correlated with pain and HAQ-DI (both 0.28; all q < 0.05). Adjusted MANSA associations remained (standardized beta = −0.49 to −0.24; all q < 0.05), with conclusions unchanged when all GSI values were included. Conclusions: Higher symptom scores were associated with poorer quality of life. Findings are exploratory and do not establish diagnostic or causal relationships.

## 1. Introduction

Rheumatoid arthritis (RA) is a chronic systemic autoimmune disease characterized by persistent synovial inflammation, progressive structural joint damage, pain, fatigue, and functional limitation. In 2021, global age-standardized RA incidence and prevalence were estimated at 11.8 and 208.9 per 100,000 population, respectively, with both rates higher in women than men [1]. RA is not ordinarily divided into epidemiologic ‘types’ for these overall burden estimates. Its impact extends beyond the joints to work ability, social functioning, family roles, emotional well-being, and quality of life [2,3,4,5].

Treat-to-target strategies and disease-modifying antirheumatic drugs have substantially improved inflammatory control [4,5]. Nevertheless, many patients continue to report pain, fatigue, and reduced function. Central pain mechanisms, illness perceptions, coping, and social circumstances may contribute to discordance between inflammatory indicators and patient-reported outcomes [6,7,8,9].

Depression and anxiety are clinically important comorbidities in RA. Systematic reviews, longitudinal cohorts, and secondary trial analyses have linked them with patient-reported disease activity, treatment response, disability, and health-related quality of life [8,10,11,12,13]. Much of this evidence comes from larger international cohorts or generic health-status measures. Evidence integrating several continuous psychological symptom domains with pain, inflammatory activity, disability, and broad subjective quality of life within Southeast European tertiary rehabilitation care remains limited.

Psychoneuroimmunological models hypothesize bidirectional relationships among inflammatory signaling, stress physiology, sleep, pain processing, emotional symptoms, and perceived health [14,15]. These models provide context but do not imply causation in a cross-sectional study. Quality-of-life assessment complements disease-activity measurement because instruments such as HAQ-DI, SF-36, EuroQol-5D, RAQoL, and MANSA represent functional or subjective domains not captured by inflammatory markers alone [8,16,17,18,19,20].

The present study contributes a systematically reported 4 × 5 continuous-score correlation matrix, false-discovery-rate control, and exploratory covariate-adjusted models from an underrepresented Bosnia and Herzegovina tertiary rehabilitation setting. Its novelty is contextual and analytic rather than causal: the design cannot determine whether psychological symptoms precede, follow, or share causes with clinical burden and quality of life.

Accordingly, we aimed to describe psychological distress, somatic symptom burden, anxiety, depressive symptoms, disease activity, functional disability, pain, and quality of life in adults with RA and to examine associations between continuous psychological scores and disease duration, pain, DAS28-CRP(3), HAQ-DI, and MANSA. We expected higher symptom scores to be associated with poorer patient-reported outcomes, while treating all analyses as exploratory.

## 2. Materials and Methods

### 2.1. Study Design and Setting

This single-center, cross-sectional observational study combined extraction of sociodemographic, historical, and treatment data from institutional medical records and the rheumatic disease registry with contemporaneous clinical and questionnaire assessment. The study was conducted at the Clinic of Physical Medicine and Rehabilitation, University Clinical Center Tuzla. Participant recruitment and research data collection commenced only after ethics approval was granted on 18 September 2025 and continued through April 2026. The study included 85 consecutive adults with RA receiving inpatient or outpatient care. The source database did not encode care setting at the participant level; therefore, the inpatient/outpatient distribution could not be reported. The disease-activity examination and questionnaires were completed on the same day. Reporting follows the STROBE statement [21].

### 2.2. Participants

Eligible participants were adults aged 20–80 years with a rheumatologist-documented RA diagnosis who fulfilled the 2010 ACR/EULAR classification criteria and had been hospitalized and/or treated as outpatients at the clinic during the study period. Age outside 20–80 years was the sole exclusion criterion. Consecutive sampling was used. All 85 eligible participants assessed during the recruitment period were included. The sample size therefore represented the available consecutive cohort, and no a priori sample-size calculation, calculation software, effect-size input, or external parameter source was documented. Direct identifiers were not processed in the analytical dataset.

### 2.3. Measures and Data Collection

Sociodemographic variables comprised age category, sex, marital status, employment status, and educational level. Income, housing, material deprivation, and other detailed socioeconomic indicators were not collected. Clinical variables comprised disease duration, morning stiffness, pain, disease activity, functional status, physical therapy participation, recorded medication category, and disease-related complications. Medication was represented by one mutually exclusive primary database category (DMARD, biologic therapy plus corticosteroid, or NSAID); dose, duration, treatment changes, adherence, concurrent medications, and whether the category was verified on the questionnaire date were not available. The ‘Other’ complication category was stored without itemized diagnoses, so its composition could not be reconstructed.

Pain intensity was assessed with a 100-mm VAS and stored on the equivalent 0–10 scale in 10-mm units. Disease activity was represented by the recorded three-component DAS28-CRP, which omits patient global assessment: DAS28-CRP(3) = [0.56 × sqrt(TJC28) + 0.28 × sqrt(SJC28) + 0.36 × ln(CRP + 1)] × 1.10 + 1.15 [22]. This version was used because the source database contained a final DAS28-CRP(3) value and no patient global component. The analytical dataset did not retain TJC28, SJC28, CRP value/date/assay, examiner identity, training or calibration, or whether the score was generated in the contemporaneous clinical record before database entry. Accordingly, the final score was analyzed as recorded and was not independently verified. Three- and four-component DAS28-CRP may differ materially for individual patients, limiting comparison with studies using DAS28-CRP(4) [23]. HAQ-DI was analyzed continuously, with higher scores indicating greater disability [16].

Psychological symptoms were assessed with the 53-item BSI, using GSI as its continuous global index [24], somatic symptom severity with PHQ-15 [25], anxiety with GAD-7 [26], and depressive symptom severity with PHQ-9 [27]. PHQ-15 items include symptoms that can arise from RA or its treatment, including pain, fatigue, sleep disturbance, and gastrointestinal complaints. Consequently, PHQ-15 was treated as a nonspecific symptom-burden measure, not as evidence of a somatic symptom disorder or as a purely psychological construct. General subjective quality of life was measured with the mean of the 12 MANSA subjective items (1–7, higher is better) [20]. MANSA was selected a priori to capture broad life domain satisfaction relevant to rehabilitation. An RA-specific measure such as RAQoL was not administered, which limits disease-specific sensitivity [19]. Nonstandard source categories for overall BSI, PST, PSDI, and MANSA were not analyzed. Thresholds of ≥10 for PHQ-15, GAD-7, and PHQ-9 are reported only as descriptive thresholds derived from original-language instruments, not locally validated diagnostic cutoffs.

All participant-reported instruments were administered in Bosnian translations used in the study setting. Documentation of forward translation, back translation, expert review, cognitive debriefing, formal local psychometric validation, and permissions where applicable was unavailable to the analytical team. Item-level responses were also unavailable, preventing calculation of Cronbach’s alpha or omega in this sample. Participants self-completed the forms with an investigator present. Investigators could clarify wording when requested but did not choose responses. The frequency and content of assistance were not recorded, so interviewer-related information bias cannot be excluded.

### 2.4. Statistical Analysis

Categorical variables are presented as frequencies and percentages. Continuous variables are summarized by mean (SD), median (IQR), and range. Shapiro–Wilk tests were performed for continuous variables and regression residuals (Supplementary File S2 (Tables S1–S9)). Several bounded or symptom variables departed from normality. Therefore, the complete 4 × 5 matrix of four psychological measures (PHQ-9, GAD-7, PHQ-15, and BSI GSI) and five clinical or quality-of-life measures (disease duration, pain VAS, DAS28-CRP(3), HAQ-DI, and MANSA) was analyzed using Spearman correlations. Benjamini–Hochberg false discovery rate (FDR) correction was applied across all 20 exact *p* values, with q < 0.05 considered statistically significant.

GSI data quality was assessed using the internal BSI relation GSI ≈ (PST × PSDI)/53. Because GSI and PSDI were commonly stored to one decimal, combined rounding could account for an absolute discrepancy of approximately 0.10. A conservative revision-stage rule flagged a record when PST was outside its permissible 0–53 range or when the absolute difference between the recorded GSI and (PST × PSDI)/53 exceeded 0.20. Four records met this rule. They were excluded only from the revised primary GSI analyses (*n* = 81). All-record GSI analyses (*N* = 85) were retained as post hoc sensitivity analyses. This rule was not prespecified. Item-level BSI responses were unavailable for auditable rescoring, and no GSI value was corrected or imputed.

Four separate exploratory linear models were fitted with standardized MANSA as the outcome and one standardized psychological score per model. Psychological measures were modeled separately because their pairwise Spearman correlations were high (rho = 0.70–0.85), precluding stable simultaneous domain-specific estimation in this modest sample. Each model adjusted for age category (indicator variables, 35–65 years reference), sex, disease duration, pain VAS, DAS28-CRP(3), and HAQ-DI, chosen to describe associations conditional on major demographic and clinical burden. Pain and HAQ-DI may be confounders, mediators, consequences, or overlapping constructs. Adjustment therefore changes the estimand and does not represent a total causal effect. Models contained nine parameters including the intercept, yielding 9.0–9.4 observations per parameter. HC3-robust standard errors and 95% CIs were used, and FDR correction was applied across four focal coefficients. Diagnostics included residual Shapiro–Wilk tests, Breusch-Pagan tests, variance inflation factors, standardized-design condition numbers, leverage, and Cook’s distance. There were no missing values; the only exclusions were the four GSI records that met the QC rule. 

Original analyses were performed using IBM SPSS Statistics, version 22.0 (IBM Corp., Armonk, NY, USA). Revised analyses were performed using Python, version 3.12.13 (Python Software Foundation, Beaverton, OR, USA), NumPy, version 2.3.5 (NumPy Developers; open-source software), and SciPy, version 1.17.0 (SciPy Developers; open-source software).

### 2.5. Artificial Intelligence (AI)-Assisted Manuscript Preparation

OpenAI ChatGPT (OpenAI, San Francisco, CA, USA, web version accessed 3 September 2026) was used for English-language editing, structural refinement, source cross-checking, reference verification, document formatting, and assistance in drafting reproducible verification code.

The authors specified the analytical plan and interpretation, and the code was executed on the de-identified database to verify descriptive statistics, correlations, FDR adjustments, regression outputs, and diagnostics. The tool was not used to generate, impute, or alter study observations. The authors reviewed all code and outputs and remain fully responsible for the analyses and manuscript.

## 3. Results

### 3.1. Sociodemographic Characteristics

The study included 85 patients with RA. Seventy participants (82.4%) were women and 15 (17.6%) were men. Fifty-one patients (60.0%) were aged 35–65 years and 30 (35.3%) were older than 65 years. Most participants were married (91.8%), and secondary education was the most frequent educational category (69.4%) (Table 1).

### 3.2. Clinical Characteristics

Mean disease duration was 12.48 years (SD 7.92), mean pain VAS was 6.16 (SD 1.18), mean DAS28-CRP(3) was 5.31 (SD 0.59), and mean HAQ-DI was 1.69 (SD 0.33). DAS28-CRP(3) values ranged from 3.32 to 6.62, with no observations below 3.32. Formal activity categories were not emphasized because the recorded three-component score is not interchangeable with DAS28-CRP(4). Intermittent or occasional physical therapy was recorded for 67 participants (78.8%). The primary medication category was DMARD for 76 (89.4%), biologic therapy plus corticosteroid for 8 (9.4%), and NSAID for 1 (1.2%) (Table 2).

### 3.3. Psychological Symptoms and Quality of Life

Mean PHQ-15, GAD-7, PHQ-9, and MANSA scores were 11.14, 7.76, 8.31, and 4.37. Among the 81 GSI records meeting the QC rule, mean GSI was 0.81 (SD 0.54). Scores ≥10 occurred in 51 participants (60.0%) on PHQ-15, 29 (34.1%) on GAD-7, and 34 (40.0%) on PHQ-9. These thresholds are descriptive and not diagnostic in this Bosnian sample (Table 3).

### 3.4. Continuous Score Correlations

Nine of 20 correlations remained significant after FDR correction. Poorer MANSA correlated with PHQ-9 (rho = −0.49, q < 0.001), GAD-7 (rho = −0.30, q = 0.016), PHQ-15 (rho = −0.37, q = 0.003), and GSI (rho = −0.53, q < 0.001). PHQ-15 correlated with pain (rho = 0.36, q = 0.004) and HAQ-DI (rho = 0.31, q = 0.016). PHQ-9 correlated with HAQ-DI (rho = 0.25, q = 0.043), and GSI correlated with pain and HAQ-DI (both rho = 0.28, q = 0.030). No psychological measure correlated significantly with disease duration or DAS28-CRP(3) after correction (Table 4). In the post hoc all-record sensitivity analysis (*N* = 85), GSI-MANSA (rho = −0.48, q < 0.001) and GSI-HAQ-DI (rho = 0.28, q = 0.028) remained significant, whereas GSI-pain did not (rho = 0.24, q = 0.059, Supplementary File S2 (Tables S1–S9)).

### 3.5. Adjusted Associations with Quality of Life

In separate exploratory models adjusted for age category, sex, disease duration, pain VAS, DAS28-CRP(3), and HAQ-DI, each psychological score was conditionally associated with poorer MANSA: PHQ-9 (standardized beta = −0.40, 95% CI −0.58 to −0.22, q < 0.001), GAD-7 (beta = −0.24, 95% CI −0.44 to −0.04, q = 0.023), PHQ-15 (beta = −0.30, 95% CI −0.57 to −0.03, q = 0.028), and GSI (beta = −0.49, 95% CI −0.65 to −0.33, q < 0.001). Adjusted R-squared ranged from 0.23 to 0.39 (Table 5). The all-record GSI sensitivity estimate was beta = −0.45 (95% CI −0.64 to −0.25, q < 0.001; adjusted R-squared = 0.37). Across primary models, maximum VIF was 1.67, standardized-design condition numbers were 2.32–2.50, residual Shapiro–Wilk *p* values were 0.26–0.62, and Breusch-Pagan *p* values were 0.10–0.44. Several observations exceeded the screening threshold Cook’s D > 4/n, but the maximum was 0.34 and none exceeded 1 (Supplementary File S2 (Tables S1–S9)).

## 4. Discussion

In this tertiary care RA sample, higher depressive, anxiety, somatic, and global psychological symptom scores were consistently associated with poorer MANSA quality of life after FDR correction. PHQ-15 and GSI were associated with pain and disability, and PHQ-9 was associated with disability. These are exploratory cross-sectional associations, not evidence of diagnostic categories, temporal direction, or treatment effects.

The depression- and anxiety-related findings are consistent with systematic reviews, prospective observational work, and trial secondary analyses linking emotional symptoms to patient-reported RA burden and subsequent physical outcomes [10,11,12,13]. Our study differs by examining a complete continuous score matrix within an underrepresented tertiary rehabilitation population and by presenting FDR-controlled and covariate-adjusted estimates. Its modest, selected sample and cross-sectional design provide less temporal and population-level information than the larger longitudinal studies.

The PHQ-15 associations with poorer MANSA, pain, and HAQ-DI require particular caution. Several PHQ-15 items overlap with RA manifestations and treatment-related symptoms, including pain, fatigue, sleep disturbance, and gastrointestinal complaints [6,7,14,15]. Associations with pain and disability may therefore partly reflect content overlap and common-method variance. PHQ-15 results in this study indicate nonspecific somatic symptom burden and cannot establish an independent somatic symptom disorder or a purely psychological mechanism.

No psychological measure correlated significantly with DAS28-CRP(3) after FDR correction. Restricted variability is one explanation: scores ranged from 3.32 to 6.62 in a clinically burdened cohort. Biological and measurement explanations are also plausible. DAS28-CRP(3) combines acute-phase inflammation and joint counts, whereas psychological scales emphasize perceived symptoms. Relationships may be nonlinear, treatment-modified, mediated through pain or fatigue, or separated by a temporal lag. Omission of patient global assessment can further reduce comparability with DAS28-CRP(4) at the individual level [23]. A same-day cross-sectional snapshot cannot distinguish these mechanisms.

The findings suggest a hypothesis for rehabilitation practice rather than proof of a screening or integrated-care intervention. Current recommendations support physical activity and self-management in inflammatory arthritis [28,29], and selected psychological or educational interventions may provide complementary benefit [30,31]. Any routine symptom assessment should use locally validated instruments, include clinical evaluation that distinguishes RA-related symptoms from mental disorders, connect positive screens to feasible referral pathways, and not substitute for rheumatologic treatment. This study did not evaluate screening accuracy, feasibility, referral capacity, intervention effects, or outcomes after referral.

The regional context is a strength because Bosnia and Herzegovina is underrepresented in RA psychosocial research. Consecutive recruitment, same-day assessment, continuous-score analysis, a complete correlation family, and transparent sensitivity analysis add rigor. The adjusted models describe associations conditional on demographic and clinical burden. They do not estimate total effects. Pain and HAQ-DI may be confounders, mediators, consequences, or overlapping constructs. Because the psychological measures were highly intercorrelated and modeled separately, the models do not establish independent domain-specific contributions beyond the other psychological scales.

Several limitations require emphasis. The cross-sectional design precludes causal or temporal inference, and no control group was available for comparison with the general population. The modest single-center sample came from inpatient and outpatient tertiary rehabilitation care, had high clinical burden and a restricted DAS28-CRP(3) range, and may not generalize to community, primary care, or less active RA populations. No a priori sample-size calculation was documented. The multivariable models had 9.0–9.4 observations per parameter and remain susceptible to limited precision and overfitting despite HC3 inference. Bosnian translations lacked documented forward and back translation, permissions, local validation, or sample-specific internal consistency because item-level data were unavailable. Investigator clarification may have introduced bias, and thresholds from the original instruments are not locally diagnostic. MANSA is a general rather than RA-specific quality-of-life measure. DAS28 component fields, assessor details, and CRP timing were unavailable, preventing recalculation. The GSI QC rule was introduced during revision, although all-record sensitivity analyses supported the main conclusion. Medication categories lacked dose, duration, changes, adherence, concurrent therapy, and confirmed contemporaneity. The ‘Other’ complication category and care setting distribution were not itemized. Employment and education were described, but income and other socioeconomic variables were absent, and socioeconomic factors were not included in the models. PHQ-15 content overlap, common-method bias, residual confounding, and multiple exploratory analyses remain important.

Future studies should use multicenter sampling, comparison groups where appropriate, prespecified outcomes and sample size calculations, longitudinal follow-up, formally translated and validated Bosnian instruments, and complete component-level disease-activity data. RA-specific quality-of-life measures, detailed socioeconomic factors, medications, comorbidity, inflammatory markers, and rehabilitation exposure would help distinguish inflammatory, functional, psychological, and contextual pathways.

## 5. Conclusions

In this tertiary care RA sample, MANSA scores correlated with depressive, anxiety, somatic, and global psychological symptom scores (rho = −0.53 to −0.30), and conditional associations remained in separate exploratory multivariable models (standardized beta = −0.49 to −0.24; all q < 0.05). PHQ-15 correlated with pain and disability, but content overlap limits psychological interpretation. The selected cross-sectional sample, unvalidated translations, general quality-of-life measure, and incomplete clinical and socioeconomic detail preclude diagnostic, causal, interventional, or population-level conclusions.

## Figures and Tables

**Table 1:** Sociodemographic characteristics of the study population (*N* = 85).

Variable	Category	*n* (%)
Age category	20–34 years	4 (4.7)
	35–65 years	51 (60.0)
	>65 years	30 (35.3)
Sex	Male	15 (17.6)
	Female	70 (82.4)
Marital status	Married	78 (91.8)
	Divorced	1 (1.2)
	Widowed	6 (7.1)
Employment status	Employed	28 (32.9)
	Unemployed	27 (31.8)
	Retired	29 (34.1)
	Disability pension	1 (1.2)
Education	University	7 (8.2)
	Secondary	59 (69.4)
	Low or no formal education	19 (22.4)

Note: Percentages use *N* = 85 as the denominator; rounding may produce totals that differ slightly from 100%.

**Table 2:** Continuous clinical measures and recorded clinical characteristics (*N* = 85).

Clinical Variable	Metric or Category	Mean (SD) or *n* (%)	Median (IQR); Range
Disease duration	Continuous, years	12.48 (7.92)	10 (8–17); 1–40
Morning stiffness	Continuous, minutes	82.35 (42.25)	60 (60–120); 10–300
Pain VAS	Continuous, 0–10	6.16 (1.18)	6 (6–7); 2–8
DAS28-CRP(3)	Continuous	5.31 (0.59)	5.37 (5.00–5.64); 3.32–6.62
HAQ-DI	Continuous, 0–3	1.69 (0.33)	1.70 (1.50–1.80); 0.80–3.00
Physical therapy	Regular	18 (21.2)	-
	Intermittent/occasional	67 (78.8)	-
Recorded medication category	DMARD	76 (89.4)	-
	Biologic therapy + corticosteroid	8 (9.4)	-
	NSAID	1 (1.2)	-
Recorded complication	Osteoporosis	30 (35.3)	-
	Other	40 (47.1)	-
	No recorded complication	15 (17.6)	-

Note: VAS = visual analogue scale; CRP = C-reactive protein; DAS28-CRP(3) = three-component 28-joint Disease Activity Score using CRP; HAQ-DI = Health Assessment Questionnaire Disability Index; DMARD = disease-modifying antirheumatic drug; NSAID = nonsteroidal anti-inflammatory drug. Medication groups were mutually exclusive primary database categories and not complete concurrent treatment histories. The ‘Other’ complication category was not itemized in the source database. Participant-level inpatient/outpatient status was not retained.

**Table 3:** Continuous psychological symptom and quality of life scores (overall *N* = 85; GSI *n* = 81).

Measure	Mean (SD)	Median (IQR)	Range	Score ≥ 10, *n* (%)
PHQ-15	11.14 (5.60)	11 (7–15)	1–25	51 (60.0)
GAD-7	7.76 (5.75)	7 (3–13)	0–21	29 (34.1)
PHQ-9	8.31 (5.67)	7 (4–12)	0–25	34 (40.0)
BSI GSI (n = 81)	0.81 (0.54)	0.80 (0.30–1.10)	0–2.30	Not categorized
MANSA	4.37 (0.82)	4.33 (3.83–4.91)	2.91–6.41	Not categorized

Note: BSI = Brief Symptom Inventory; GSI = Global Severity Index; MANSA = Manchester Short Assessment of Quality of Life. Thresholds ≥10 are descriptive only and are not locally validated diagnostic cutoffs. GSI excludes four records meeting the revision-stage QC rule.

**Table 4:** Complete matrix of Spearman correlations using continuous scores (overall *N* = 85; GSI analyses *n* = 81).

Psychological Measure	Clinical/QoL Measure	*n*	Rho	*p* Value	q Value
PHQ-9	Disease duration	85	0.21	0.056	0.080
PHQ-9	Pain VAS	85	0.20	0.070	0.093
PHQ-9	DAS28-CRP(3)	85	0.10	0.351	0.390
PHQ-9	HAQ-DI	85	**0.25**	**0.019**	**0.043**
PHQ-9	MANSA	85	**−0.49**	**<0.001**	**<0.001**
GAD-7	Disease duration	85	0.13	0.219	0.257
GAD-7	Pain VAS	85	0.22	0.046	0.071
GAD-7	DAS28-CRP(3)	85	0.02	0.870	0.870
GAD-7	HAQ-DI	85	0.14	0.215	0.257
GAD-7	MANSA	85	**−0.30**	**0.005**	**0.016**
PHQ-15	Disease duration	85	0.23	0.036	0.065
PHQ-15	Pain VAS	85	**0.36**	**<0.001**	**0.004**
PHQ-15	DAS28-CRP(3)	85	0.22	0.042	0.069
PHQ-15	HAQ-DI	85	**0.31**	**0.004**	**0.016**
PHQ-15	MANSA	85	**−0.37**	**<0.001**	**0.003**
BSI GSI	Disease duration	81	0.06	0.588	0.619
BSI GSI	Pain VAS	81	**0.28**	**0.012**	**0.030**
BSI GSI	DAS28-CRP(3)	81	0.24	0.028	0.056
BSI GSI	HAQ-DI	81	**0.28**	**0.012**	**0.030**
BSI GSI	MANSA	81	**−0.53**	**<0.001**	**<0.001**

Note: BSI = Brief Symptom Inventory; GSI = Global Severity Index. q values were calculated across all 20 correlations using exact *p* values. Bold indicates q < 0.05. Four internally discordant records were excluded only from primary GSI analyses; all-record sensitivity results appear in Supplementary File S2 (Tables S1–S9).

**Table 5:** Separate adjusted linear models of continuous MANSA quality of life.

Psychological Predictor	*n*	Standardized Beta	95% CI	*p* Value	q Value	Adjusted R-Squared
PHQ-9	85	−0.40	−0.58 to −0.22	<0.001	<0.001	0.33
GAD-7	85	−0.24	−0.44 to −0.04	0.017	0.023	0.23
PHQ-15	85	−0.30	−0.57 to −0.03	0.028	0.028	0.25
BSI GSI	81	−0.49	−0.65 to −0.33	<0.001	<0.001	0.39

Note: Each row is a separate exploratory model with standardized MANSA and one standardized psychological predictor. Covariates were age category (35–65 years reference), sex, disease duration, pain VAS, DAS28-CRP(3), and HAQ-DI. HC3-robust standard errors were used; q values reflect FDR adjustment across the four focal coefficients. Full unstandardized models and diagnostics are in Supplementary File S2 (Tables S1–S9). CI = confidence interval.

## Data Availability

The anonymized data supporting the findings of this study are available from the corresponding author upon reasonable request, subject to institutional and ethical restrictions.

## Supplementary Materials

The following supporting information can be downloaded at: https://www.mdpi.com/article/10.3390/healthcare14193179/s1, The following supporting information is available with this article: Supplementary File S1: Updated STROBE checklist for a cross-sectional study; Supplementary File S2: Expanded statistical results, diagnostics, and GSI sensitivity analyses, comprising (Table S1: Shapiro-Wilk tests for continuous analysis variables; Table S2: Intercorrelations among psychological measures; Table S3: Full HC3 regression model with PHQ-9 as focal predictor; Table S4: Full HC3 regression model with GAD-7 as focal predictor; Table S5: Full HC3 regression model with PHQ-15 as focal predictor; Table S6: Full HC3 regression model with BSI GSI as focal predictor; Table S7: Regression model diagnostics; Table S8: Primary and post hoc all-record GSI correlation comparisons; Table S9: Primary and post hoc all-record GSI regression comparisons.

## Abbreviations

The following abbreviations are used in this manuscript:

ACR	American College of Rheumatology
AI	Artificial intelligence
BSI	Brief Symptom Inventory
CI	Confidence interval
CRP	C-reactive protein
DAS28-CRP(3)	Three-component 28-joint Disease Activity Score using C-reactive protein
DMARD	Disease-modifying antirheumatic drug
EULAR	European Alliance of Associations for Rheumatology
FDR	False discovery rate
GAD-7	Generalized Anxiety Disorder-7
GH/PGA	Global health/patient global assessment
GSI	Global Severity Index
HAQ-DI	Health Assessment Questionnaire Disability Index
HC3	Heteroscedasticity-consistent covariance estimator, type 3
MANSA	Manchester Short Assessment of Quality of Life
NSAID	Nonsteroidal anti-inflammatory drug
PHQ-9	Patient Health Questionnaire-9
PHQ-15	Patient Health Questionnaire-15
PSDI	Positive Symptom Distress Index
PST	Positive Symptom Total
RA	Rheumatoid arthritis
SJC28	28-joint swollen-joint count
SPSS	Statistical Package for the Social Sciences
STROBE	Strengthening the Reporting of Observational Studies in Epidemiology
TJC28	28-joint tender-joint count
VAS	Visual analogue scale
