# Supplementary material for: Psychological Distress, Somatic Symptom Burden, and Quality of Life in Rheumatoid Arthritis: A Cross-Sectional Study in Bosnia and Herzegovina
Source: Healthcare (Basel). 2026 Sep 25;14(19):3179. doi: 10.3390/healthcare14193179 (PMC13665265; doi:10.3390/healthcare14193179)
Supplement: Supplementary file 1 [file healthcare-14-03179-s001.zip › Supplementary File S1 STROBE Checklist final.pdf]

SUPPLEMENTARY FILE S1

Updated STROBE checklist for a cross-sectional study

Manuscript ID: healthcare-4508597

This checklist maps the final revised manuscript to the STROBE reporting items. Wording below is a concise paraphrase of the checklist recommendations.

Item	Reporting element	Location in revised manuscript
**1a**	Identify the design in the title or abstract.	Title; Abstract—Methods
**1b**	Provide an informative, balanced abstract.	Abstract
**2**	Explain scientific background and rationale.	Introduction, paragraphs 1–5
**3**	State specific objectives and hypotheses.	Introduction, final paragraph
**4**	Present key study-design elements early.	Section 2.1
**5**	Describe setting, location, and dates.	Section 2.1
**6a**	Give eligibility criteria and participant selection.	Section 2.2
**6b**	Matching criteria for matched studies.	Not applicable; unmatched single-group study
**7**	Define outcomes, exposures, predictors, confounders, and effect modifiers.	Sections 2.3–2.4
**8**	Give data sources and measurement methods.	Section 2.3
**9**	Describe efforts to address bias.	Consecutive recruitment and same-day assessment: Sections 2.1–2.2; measurement and residual biases: Sections 2.3 and 4
**10**	Explain how study size was determined.	Section 2.2; Limitations
**11**	Explain handling of quantitative variables and groupings.	Sections 2.3–2.4; Tables 2–3
**12a**	Describe statistical methods and confounding control.	Section 2.4; Supplementary File S2
**12b**	Describe subgroup and interaction methods.	No subgroup or interaction analyses performed
**12c**	Explain missing-data handling.	Section 2.4; no missing values; four GSI records excluded only from primary GSI analyses under the QC rule

Item	Reporting element	Location in revised manuscript
**12d**	Address sampling strategy where relevant.	Consecutive sample described in Section 2.2; no survey weighting
**12e**	Describe sensitivity analyses.	Section 2.4; Sections 3.4–3.5; Supplementary File S2 (post hoc sensitivity analyses)
**13a**	Report numbers at each participation stage.	Section 2.2; all 85 participants included; primary GSI analyses n = 81 after record-level QC
**13b**	Give reasons for non-participation.	No non-participation recorded among the eligible consecutive cohort
**13c**	Consider a flow diagram.	Not included; no participant exclusions after eligibility; four GSI records excluded only from primary GSI analyses
**14a**	Report participant and exposure characteristics.	Sections 3.1–3.3; Tables 1–3
**14b**	Report missing data for variables.	Section 2.4; no missing values; Tables 3–5 report GSI n = 81 after QC exclusions
**14c**	Summarize follow-up time where applicable.	Not applicable; cross-sectional assessment
**15**	Report outcome data.	Sections 3.2–3.3; Tables 2–3
**16a**	Give unadjusted and adjusted estimates with precision.	Sections 3.4–3.5; Tables 4–5; Supplementary File S2
**16b**	Report category boundaries when continuous variables are categorized.	Sections 2.3 and 3.3; Table 3 note; thresholds descriptive only
**16c**	Translate relative risks when relevant.	Not applicable; no relative-risk estimates
**17**	Report other analyses, including sensitivity analyses.	Sections 3.4–3.5; Supplementary File S2
**18**	Summarize key results with reference to objectives.	Discussion, paragraph 1
**19**	Discuss study limitations and likely bias direction/magnitude.	Discussion, limitations paragraph
**20**	Provide cautious overall interpretation.	Discussion; Conclusions
**21**	Discuss generalizability.	Discussion and Limitations
**22**	State funding and funder role.	Funding statement
