# Supplementary material for: Psychological Distress, Somatic Symptom Burden, and Quality of Life in Rheumatoid Arthritis: A Cross-Sectional Study in Bosnia and Herzegovina
Source: Healthcare (Basel). 2026 Sep 25;14(19):3179. doi: 10.3390/healthcare14193179 (PMC13665265; doi:10.3390/healthcare14193179)
Supplement: Supplementary file 1 [file healthcare-14-03179-s001.zip › Supplementary File S2 Statistical Results final revision.pdf]

SUPPLEMENTARY FILE S2

Expanded statistical results, diagnostics, and GSI sensitivity analyses

Manuscript ID: healthcare-4508597

All analyses use continuous scores. HC3 inference uses t distributions with residual degrees of freedom. No recorded source value was corrected or imputed.

Table S1. Shapiro-Wilk tests for continuous analysis variables

Variable	N	W	p value
**Disease duration**	85	0.908	<0.001
**Pain VAS**	85	0.857	<0.001
**DAS28-CRP(3)**	85	0.978	0.155
**HAQ-DI**	85	0.936	<0.001
**MANSA**	85	0.977	0.133
**PHQ-9**	85	0.935	<0.001
**GAD-7**	85	0.930	<0.001
**PHQ-15**	85	0.972	0.063
**BSI GSI**	81	0.911	<0.001

Several bounded or symptom variables departed from normality, supporting rank-based bivariate analyses.

Table S2. Intercorrelations among psychological measures

Measure 1	Measure 2	N	Spearman rho	p value
**PHQ-9**	GAD-7	85	0.85	<0.001
**PHQ-9**	PHQ-15	85	0.79	<0.001
**PHQ-9**	BSI GSI	81	0.76	<0.001
**GAD-7**	PHQ-15	85	0.73	<0.001
**GAD-7**	BSI GSI	81	0.70	<0.001
**PHQ-15**	BSI GSI	81	0.78	<0.001

The high correlations (rho = 0.70–0.85) motivated separate exploratory models and precluded claims of domain-specific independence.

Table S3. Full HC3 regression model with PHQ-9 as focal predictor

Term	B	HC3 SE	95% CI	p value
**Intercept**	5.049	0.885	3.287 to 6.811	<0.001
**PHQ-9**	-0.058	0.013	-0.084 to -0.031	<0.001
**Age 20–34 vs 35–65**	0.984	0.664	-0.338 to 2.306	0.142
**Age >65 vs 35–65**	-0.390	0.155	-0.699 to -0.081	0.014
**Female vs male**	-0.371	0.176	-0.721 to -0.021	0.038
**Disease duration, years**	0.001	0.011	-0.020 to 0.023	0.895
**Pain VAS, 0–10**	0.068	0.084	-0.099 to 0.236	0.420
**DAS28-CRP(3)**	0.008	0.171	-0.333 to 0.349	0.963
**HAQ-DI**	-0.168	0.245	-0.656 to 0.319	0.494

N = 85; nine parameters including the intercept; residual df = 76; R-squared = 0.397; adjusted R-squared = 0.333. Outcome is MANSA in its original units; coefficients are unstandardized.

Table S4. Full HC3 regression model with GAD-7 as focal predictor

Term	B	HC3 SE	95% CI	p value
**Intercept**	5.320	0.925	3.477 to 7.163	<0.001
**GAD-7**	-0.035	0.014	-0.063 to -0.006	0.017
**Age 20–34 vs 35–65**	0.995	0.599	-0.199 to 2.189	0.101
**Age >65 vs 35–65**	-0.441	0.169	-0.778 to -0.104	0.011
**Female vs male**	-0.426	0.197	-0.818 to -0.034	0.033
**Disease duration, years**	-0.001	0.011	-0.024 to 0.021	0.896
**Pain VAS, 0–10**	0.092	0.091	-0.089 to 0.274	0.314
**DAS28-CRP(3)**	-0.060	0.180	-0.419 to 0.299	0.741
**HAQ-DI**	-0.270	0.280	-0.827 to 0.287	0.338

N = 85; nine parameters including the intercept; residual df = 76; R-squared = 0.307; adjusted R-squared = 0.234. Outcome is MANSA in its original units; coefficients are unstandardized.

Table S5. Full HC3 regression model with PHQ-15 as focal predictor

Term	B	HC3 SE	95% CI	p value
**Intercept**	4.960	0.955	3.059 to 6.861	<0.001
**PHQ-15**	-0.044	0.019	-0.082 to -0.005	0.028
**Age 20–34 vs 35–65**	1.001	0.782	-0.556 to 2.559	0.204
**Age >65 vs 35–65**	-0.394	0.159	-0.711 to -0.078	0.015
**Female vs male**	-0.345	0.218	-0.779 to 0.089	0.118
**Disease duration, years**	-0.001	0.012	-0.026 to 0.024	0.922
**Pain VAS, 0–10**	0.121	0.100	-0.079 to 0.321	0.232
**DAS28-CRP(3)**	-0.026	0.178	-0.380 to 0.328	0.885
**HAQ-DI**	-0.190	0.279	-0.746 to 0.365	0.497

N = 85; nine parameters including the intercept; residual df = 76; R-squared = 0.321; adjusted R-squared = 0.249. Outcome is MANSA in its original units; coefficients are unstandardized.

Table S6. Full HC3 regression model with BSI GSI as focal predictor

Term	B	HC3 SE	95% CI	p value
**Intercept**	4.471	0.821	2.834 to 6.107	<0.001
**BSI GSI**	-0.741	0.123	-0.986 to -0.495	<0.001
**Age 20–34 vs 35–65**	0.991	0.672	-0.348 to 2.331	0.144
**Age >65 vs 35–65**	-0.363	0.143	-0.648 to -0.077	0.013
**Female vs male**	-0.267	0.180	-0.625 to 0.092	0.143
**Disease duration, years**	-0.005	0.011	-0.028 to 0.018	0.680
**Pain VAS, 0–10**	0.127	0.079	-0.031 to 0.285	0.114
**DAS28-CRP(3)**	0.057	0.153	-0.248 to 0.361	0.712
**HAQ-DI**	-0.114	0.222	-0.556 to 0.328	0.610

N = 81; nine parameters including the intercept; residual df = 72; R-squared = 0.455; adjusted R-squared = 0.395. Outcome is MANSA in its original units; coefficients are unstandardized.

Table S7. Regression model diagnostics

Focal predictor	N	Obs./param.	Max VIF	Cond. no.	SW p	BP p	Max Cook D	Cook's D > 4/n

Focal predictor	N	Obs./param.	Max VIF	Cond. no.	SW p	BP p	Max Cook D	Cook's D > 4/n
**PHQ-9**	85	9.4	1.55	2.32	0.428	0.205	0.20	7
**GAD-7**	85	9.4	1.59	2.37	0.261	0.443	0.12	6
**PHQ-15**	85	9.4	1.67	2.50	0.394	0.251	0.34	6
**BSI GSI**	81	9.0	1.55	2.33	0.624	0.100	0.22	8

SW = Shapiro-Wilk test of residuals; BP = Breusch-Pagan test; VIF = variance inflation factor. Condition numbers use a standardized design. No Cook's D exceeded 1. Leverage and predictor-specific VIF values were also inspected; no numerical singularity was found. These checks do not eliminate small-sample overfitting risk.

Table S8. Primary and post hoc all-record GSI correlation comparisons

Outcome	Primary rho (n = 81)	Primary q	All-record rho (n = 85)	All-record q
**Disease duration**	0.06	0.619	0.03	0.862
**Pain VAS**	0.28	0.030	0.24	0.059
**DAS28-CRP(3)**	0.24	0.056	0.21	0.077
**HAQ-DI**	0.28	0.030	0.28	0.028
**MANSA**	-0.53	<0.001	-0.48	<0.001

QC rule: records were excluded from primary GSI analyses if PST was outside 0–53 or if |recorded GSI − (PST × PSDI)/53| exceeded 0.20. Four records met this post hoc revision-stage rule. Item-level responses were unavailable for auditable rescoring; no value was altered or imputed. The all-record sensitivity analysis retained all 85 GSI values. Correlation q values were calculated separately within each 20-correlation family using exact p values.

Table S9. Primary and post hoc all-record GSI regression comparisons

Analysis	N	Standardized beta	95% CI	q value	Adjusted R-squared
**Primary QC-eligible GSI analysis**	81	-0.49	-0.65 to -0.33	<0.001	0.39
**Post hoc all-record GSI analysis**	85	-0.45	-0.64 to -0.25	<0.001	0.37

For each analysis, q values reflect FDR adjustment across the four focal coefficients. Both analyses used the same covariate set described in the main manuscript.
